# Impact of hemodialysis on efficacies of the antiplatelet agents in coronary artery disease patients complicated with end-stage renal disease

**DOI:** 10.1007/s11239-023-02924-5

**Published:** 2024-02-23

**Authors:** Zekang Ye, Qin Wang, Inam Ullah, Qingxia Lin, Tianyu Wu, Mingwen Yang, Yuansheng Fan, Zhou Dong, Tong Wang, Jianzhen Teng, Rui Hua, Yingdan Tang, Yule Li, Xiaoxuan Gong, Liang Yuan, Zhengxian Tao, Chunjian Li

**Affiliations:** 1 Department of Cardiology, The First Affiliated Hospital of Nanjing Medical University, Nanjing, Jiangsu China; 2 Department of Neurology, The Fifth Affiliated Hospital of Wenzhou Medical University, Lishui, Zhejiang China; 3 Department of Biostatistics, School of Public Health, Nanjing Medical University, Nanjing, Jiangsu China; 4 College of Letters and Science, University of Wisconsin-Madison, Madison, WI USA

**Keywords:** Coronary artery disease, End-stage renal disease, Hemodialysis, Antiplatelet therapy

## Abstract

It is controversial whether hemodialysis affects the efficacy of the antiplatelet agents. We aimed to investigate the impact of hemodialysis on efficacies of the antiplatelet agents in coronary artery disease (CAD) patients complicated with end-stage renal disease (ESRD). 86 CAD patients complicated with ESRD requiring hemodialysis were consecutively enrolled. After 5-day treatment with aspirin and clopidogrel or ticagrelor, the platelet aggregations induced by arachidonic acid (PL_AA_) or adenosine diphosphate (PL_ADP_), and the P2Y_12_ reaction unit (PRU) were measured before and after hemodialysis. The propensity matching score method was adopted to generate a control group with normal renal function from 2439 CAD patients. In patients taking aspirin, the PL_AA_ remained unchanged after hemodialysis. In patients taking clopidogrel, the PL_ADP_ (37.26 ± 17.04 vs. 31.77 ± 16.09, *p* = 0.029) and corresponding clopidogrel resistance (CR) rate (23 [48.9%] vs. 14 [29.8%], *p* = 0.022) significantly decreased after hemodialysis, though PRU remained unchanged. Subgroup analysis indicated that PL_ADP_ significantly decreased while using polysulfone membrane (36.8 ± 17.9 vs. 31.1 ± 14.5, *p* = 0.024). In patients taking ticagrelor, PL_ADP_, and PRU remained unchanged after hemodialysis. ESRD patients had higher incidences of aspirin resistance (AR) and CR compared to those with normal renal function (AR: 16.1% vs. 0%, *p* = 0.001; CR: 48.4% vs. 24.8%, *p* = 0.024). Hemodialysis does not have negative effect on the efficacies of aspirin, clopidogrel and ticagrelor in ESRD patients with CAD. ESRD patients have higher incidences of AR and CR compared with those with normal renal function.

*Trial registration* ClinicalTrials.gov Identifier: NCT03330223, first registered January 4, 2018.

**Supplementary Information:**

The online version contains supplementary material available at 10.1007/s11239-023-02924-5.

## Highlights

- The impact of hemodialysis on the efficacies of antiplatelet agents is uncertain.
- Hemodialysis did not affect the efficacies of aspirin and ticagrelor.
- Hemodialysis using polysulfone membrane might improve the clopidogrel response.
- Patients with ESRD had higher incidences of aspirin and clopidogrel resistance.

## Introduction

Patients with chronic kidney disease, especially those in end-stage renal disease (ESRD), have a higher incidence of coronary artery disease (CAD) than the general population [1]. According to the US Renal Data System 2019 Annual Data Report [2, 3], the number of ESRD patients undergoing percutaneous coronary intervention (PCI) increases by almost 50% compared with general population [4].

However, previous large clinical trials evaluating optimal antiplatelet therapy have historically excluded hemodialysis patients because of their high rate of cardiovascular events, high non-cardiovascular mortality, and increased case-fatality, this might have skewed the overall results and the power to detect a therapeutic effect, and leaves a gap in existing knowledge [5, 6].

The chronic kidney disease was reported to be independent predictor of stent thrombosis in patients undergoing PCI therapy [7, 8], which might be associated with weakened efficacy of the antiplatelet agents [9–11]. ESRD patients have to receive hemodialysis after several years, and it is controversial whether hemodialysis has an effect on the efficacies of the antiplatelet agents [12, 13].

This study was to investigate the impact of hemodialysis on efficacies of the antiplatelet agents in CAD patients complicated with ESRD.

## Methods

### Study design

This is a single-center, open-label study to evaluate the effect of hemodialysis on the antiplatelet effects of aspirin, clopidogrel and ticagrelor in patients with CAD and ESRD. Subjects were enrolled from January 4, 2018, to March 29, 2022 in the First Affiliated Hospital of Nanjing Medical University. This study complied with the Declaration of Helsinki (64th, 2013) and was approved by the Ethics Committee of the First Affiliated Hospital of Nanjing Medical University and was registered at www.clinicaltrials.gov (Unique Identifier: NCT03330223). The informed consent was acquired from each participants.

### Study subjects

Patients with CAD and ESRD were consecutively recruited if they met the following inclusion criteria: (1) taking dual antiplatelet drugs (aspirin 100 mg per day plus clopidogrel 75 mg per day or ticagrelor 90 mg twice daily) for at least 5 days; (2) requiring hemodialysis as renal replacement therapy. Exclusion criteria were patients: (1) intolerant to aspirin, clopidogrel or ticagrelor; (2) with abnormal baseline platelet counts of < 100 × 10^9^/L or > 450 × 10^9^/L; (3) taking nonsteroidal anti-inflammatory drugs other than aspirin; (4) using IIb/IIIa inhibitors in the past 10 days; (5) with history of hemorrhagic disease; (6) with cancer or any other complications that may not be suitable to be included at the discretion of the investigators. In this study, the population with CAD was defined as patients given a diagnosis of stable angina, unstable angina, or myocardial infarction or people who had a coronary intervention such as percutaneous stent insertion.

### Blood sample collection

Venous blood was collected into two 2.7 mL and one 2.0 mL vacutainer tubes containing 0.105 M buffered sodium citrate (3.2%) for LTA and VerifyNow assays respectively. The samples were collected within 10 min before and after hemodialysis. Platelet function assays were completed within 3 h of blood collection.

### Platelet function tests

#### Light transmission aggregation (LTA)

Platelet function was analyzed using a Chrono-log Model 700 aggregometer (Chrono-log Corporation, Havertown, PA). Platelet-rich plasma (PRP) and platelet-poor plasma (PPP) were prepared shortly after blood collection by spinning the sample at 200×*g* for 5 min in the centrifuge machine. The PRP was carefully removed, and the remaining blood was centrifuged at 2465×*g* for 10 min to obtain PPP. The centrifuge temperature was maintained at 22 °C. Platelet counts were adjusted by adding PPP to the PRP to achieve a count of 250 × 10^9^/L. Then, 500 μL adjusted PRP was transferred into a test tube, and a 500 μL PPP was set as a control. Arachidonic acid induced platelet aggregation (PL_AA_) and adenosine diphosphate induced platelet aggregation (PL_ADP_) were recorded within 8 min [14]. Aspirin resistance was defined as PL_AA_ > 20%, and clopidogrel or ticagrelor resistance was defined as PL_ADP_ > 40% [15, 16].

### VerifyNow P2Y_12_ assay

Platelet function was also analyzed using a VerifyNow system (Accumetrics, San Diego, CA). The VerifyNow P2Y_12_ test cartridge measures platelet aggregation in separate channels in response to ADP, and thrombin receptor activating peptide (TRAP) as a reference. Results from the ADP channel are reported as P2Y_12_ reactivity units (PRU). Clopidogrel resistance (CR) or ticagrelor resistance (TR) was defined as PRU > 208 [17, 18].

### Propensity score matching

To investigate the difference of CR in ESRD patients and those with normal renal function, propensity score matching was performed using a pre-registered cohort of 2439 patients with acute coronary syndrome or stable coronary artery disease undergoing coronary stent implantation and receiving aspirin and clopidogrel in the First Affiliated Hospital of Nanjing Medical University, Nanjing, China, between January 2011 and September 2016 [19]. The clinical characteristics of these are shown in Supplementary Table 1. SAS software, version 9.4 (SAS Institute Inc., Cary, NC, USA) was adopted to generate a control group with normal renal function from the cohort according to 1:4 ratio. The efficacies of clopidogrel in patients with ESRD and those with normal renal function was compared.

### Statistical analysis

Statistical analysis was performed using SAS software, version 9.4 (SAS Institute Inc., Cary, NC, USA). Continuous variables were expressed as mean ± standard deviation (SD) or median with interquartile range (IQR) as appropriate. Categorical variables were presented as frequencies. The primary pharmacodynamic parameter was PL_AA_ for aspirin and PL_ADP_ for P2Y_12_ inhibitors (clopidogrel and ticagrelor). The secondary pharmacodynamic parameter was PRU for P2Y_12_ inhibitors. Paired-sample *t* test and McNemar’s test was used to compare the pharmacodynamic parameters of antiplatelet agents before and after hemodialysis. Student *t* test and chi-square test were used to compare baseline characteristics and platelet functions between ESRD patients and those with normal renal function. All statistical tests were two-tailed at a α level of 0.05 for significance.

## Results

### Clinical characteristics

A total of 86 patients with CAD and ESRD on hemodialysis were included. Of the included patients, 47 were treated with aspirin combined with clopidogrel, and 39 were treated with aspirin combined with ticagrelor. The clinical characteristics of the patients are shown in Table 1.

**Table 1:** Baseline characteristics of the recruited patients

Characteristics	All subjects n = 86	Clopidogrel n = 47	Ticagrelor n = 39
Age (years; mean ± SD)	67.2 ± 10.5	70.3 ± 9.5	63.5 ± 10.4
Gender (n, %)	22 (25.6%)	12 (25.5%)	10 (25.6%)
BMI (kg/m^2^)	23.8 ± 3.6	23.9 ± 3.2	23.7 ± 4.1
Hypertension (n, %)	73 (84.9%)	38 (80.9%)	35 (89.7%)
Diabetes (n, %)	51 (60.0%)	32 (69.6%)	19 (48.7%)
Smoking (n, %)	28 (32.9%)	15 (32.6%)	13 (33.3%)
Alcohol (n, %)	17 (20.0%)	12 (26.1%)	5 (12.8%)
WBC (× 10^9^/L)	7.6 ± 3.4	7.9 ± 3.7	7.1 ± 2.9
Platelet (× 10^9^/L)	165.8 ± 69.0	173.7 ± 78.1	156.6 ± 56.3
Hb (g/L)	97.8 ± 21.4	93.4 ± 21.8	102.8 ± 20.0
LDL (mmol/L)	2.1 ± 0.9	2.2 ± 0.9	2.1 ± 0.9
HDL (mmol/L)	0.9 ± 0.3	0.9 ± 0.2	0.8 ± 0.3
TC (mmol/L)	3.5 ± 1.3	3.6 ± 1.1	3.3 ± 1.4
TG (mmol/L)	1.7 ± 2.2	1.4 ± 0.9	1.9 ± 3.0
Uric acid (μmol/L)	332.8 ± 145.3	318.1 ± 135.8	353.9 ± 150.9
Creatine (μmol/L)	630.1 ± 408.1	557.6 ± 255.2	720.4 ± 526.4
Ejection fraction (%)	52.9 ± 9.8	54.8 ± 8.2	51.3 ± 11.0
Diagnosis			
SA (n, %)	22 (25.6%)	12 (25.5%)	10 (25.6%)
ACS (n, %)	64 (74.4%)	35 (74.5%)	29 (74.4%)
Concomitant medications			
Statins (n, %)	79 (94.0%)	42 (91.3%)	37 (97.4%)
PPIs (n, %)	45 (53.6%)	26 (56.5%)	19 (50.0%)
Nitrates (n, %)	68 (80.0%)	37 (80.4%)	31 (79.5%)
β blockers (n, %)	68 (80.0%)	37 (80.4%)	31 (79.5%)
CCB (n, %)	46 (54.1%)	22 (48.9%)	24 (61.5%)
ACEI/ARB (n, %)	25 (29.1%)	13 (28.9%)	12 (30.8%)

*BMI* body mass index, *WBC* white blood cell, *Hb* hemoglobin, *LDL* low density lipoprotein, *HDL* high density lipoprotein, *TC* total cholesterol, *TG* triglyceride, *SA* stable angina, *ACS* acute coronary syndrome, *PPIs* proton pump inhibitors, *CCB* calcium channel blockers, *ACEI/ARB* angiotensin-converting enzyme inhibitor and antagonist

Three types of filters were adopted in this study as follows: AV600s (Fresenius Medical Care, Bad Homburg, Germany), HF1200 (Medivators inc., Minneapolis, MN, USA), and 14L (Baxter Healthcare, Deerfield, Illinois, USA). The AV600s and HF1200 filters were made of polysulfone membrane, and the 14L filter was made of polyamide membrane. Different types of the filters were chosen at the discretion of the attending physicians.

Totally 108 hemodialysis were performed for the included patients, in which two hemodialysis sessions were performed in 22 patients using different types of membrane i. e, polysulfone and polyamide membranes.

### Platelet aggregation after hemodialysis

**Fig. 1:**
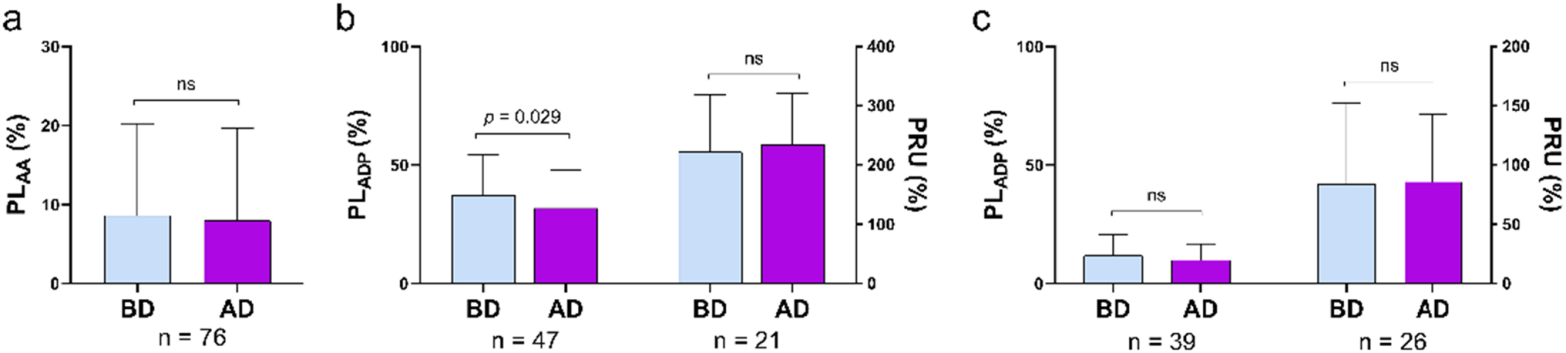
Platelet activities on different antiplatelet agents before and after hemodialysis. PL_AA_ represents the pharmacological effects of aspirin (**a**); PL_ADP_ and PRU reflect the activity of the P2Y_12_ receptor and represent the pharmacological effects of clopidogrel (**b**) and ticagrelor (**c**). *BD* before dialysis, *AD* after dialysis, *PL*_*AA*_ The maximum platelet aggregation rate induced by arachidonic acid, *PL*_*ADP*_ The maximum platelet aggregation rate induced by adenosine diphosphate, *PRU* P2Y_12_ reaction unit

For patients taking aspirin, no significant differences were observed in PL_AA_ after hemodialysis (Fig. 1a). For patients taking clopidogrel, PL_ADP_ was significantly decreased after hemodialysis (37.26 ± 17.04 *vs*. 31.77 ± 16.09, *p* = 0.029), though there was no significant change in PRU (Fig. 1b). For patients taking ticagrelor, there was no significant change in PL_ADP_, or PRU after hemodialysis (Fig. 1c).

No significant change in the incidence of drug resistance was observed after hemodialysis for aspirin regardless of the assay used (Table 2). The incidence of CR significantly decreased after hemodialysis based on the result of the LTA assay (23 [48.9%] *vs.* 14 [29.8%], *p* = 0.022). Though the PRU remained unchanged, it showed a tendency of decrease towards significant difference (12 [57.1%] *vs.* 7 [33.1%], *p* = 0.063) (Table 2). It should be noted that no ticagrelor resistance occurred in patients taking ticagrelor before or after hemodialysis by LTA assay. Only two patients presented ticagrelor resistance before and after hemodialysis respectively by VeryfyNow assay (Table 2).

**Table 2:** Comparison of the incidences of antiplatelet drug resistance before and after hemodialysis

Drug resistance	Before hemodialysis	After hemodialysis	*p* value
AR by PL_AA_	9 (11.8%)	7 (9.2%)	0.727
CR by PL_ADP_	23 (48.9%)	14 (29.8%)	0.022
CR by PRU	12 (57.1%)	7 (33.3%)	0.063
TR by PL_ADP_	0 (0%)	0 (0%)	–
TR by PRU	1 (3.8%)	1 (3.8%)	1.000

Values are presented as n (%). AR was defined as a PL_AA_ of > 20%. CR by PL_ADP_ and TR by PL_ADP_ was defined as a PL_ADP_ of > 40%. CR by PRU and TR by PRU was defined as a PRU of > 208

*AR* aspirin resistance, *CR* clopidogrel resistance, *TR* ticagrelor resistance, *PL*_*AA*_ arachidonic acid–induced platelet aggregation, *PL*_*ADP*_ ADP-induced platelet aggregation, *PRU* P2Y_12_ reactivity units

### Effect of different membrane materials on the efficacies of the antiplatelet agents

**Fig. 2:**
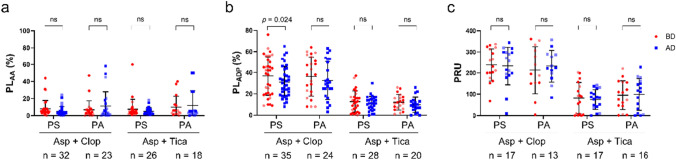
Platelet activities in different groups before and after hemodialysis with different membrane materials. *PL*_*AA*_ represents the pharmacological effects of aspirin (**a**) before and after hemodialysis with polysulfone or polyamide membrane; *PL*_*ADP*_ and PRU  represent the pharmacological effects of clopidogrel (**b**) and ticagrelor (**c**) before and after hemodialysis with polysulfone or polyamide membrane. *Asp* aspirin, *Clop* clopidogrel, *Tica* ticagrelor, *PS* polysulfone membrane, *PA* polyamide membrane, *BD* before dialysis, *AD* after dialysis, *PL*_*AA*_ the maximum platelet aggregation rate induced by arachidonic acid, *PL*_*ADP*_ the maximum platelet aggregation rate induced by adenosine diphosphate, *PRU* P2Y_12_ reaction unit

In patients taking aspirin and clopidogrel, polysulfone membrane was used in 42 hemodialysis, and polyamide membrane was used in 21 hemodialysis. In patients taking aspirin and ticagrelor, polysulfone membrane was used in 27 hemodialysis, and polyamide membrane was used in 18 hemodialysis. PL_AA_ remained unchanged after hemodialysis regardless of the membrane used (Fig. 2a).

In patients taking clopidogrel, PL_ADP_ significantly decreased after hemodialysis with polysulfone membrane (36.8 ± 17.9 *vs.* 31.1 ± 14.5, *p* = 0.024) (Fig. 2b). However, no significant change in PL_ADP_ was observed while polyamide membrane was adopted (Fig. 2b) and no significant change in PRU was observed after hemodialysis regardless of the membrane used (Fig. 2c).

Further analysis showed that while polysulfone membrane was adopted in hemodialysis for patients taking clopidogrel, 26 patients remained CR after hemodialysis, 9 changed from CR to non-resistance (Table 3). The incidence of CR was significantly decreased after hemodialysis (51.4% *vs.* 25.7%, *p* = 0.004).

**Table 3:** CR in patients taking clopidogrel undergoing hemodialysis with polysulfone membrane

		After hemodialysis		Total
		Non-resistance	Resistance	
Before hemodialysis	Non-resistance	17 (48.6%)	0 (0%)	17 (48.6%)
	Resistance	9 (25.7%)	9 (25.7%)	18 (51.4%)
	Total	26 (74.3%)	9 (25.7%)	35 (100%)

Values are expressed as n (%)

*CR* clopidogrel resistance

In patients taking ticagrelor, both PL_ADP_ and PRU remained unchanged after hemodialysis regardless of the hemodialysis membrane used (Fig. 2c).

### Clopidogrel responses between ESRD patients and those with normal renal function

By propensity score matching to minimize the differences of baseline characteristics, 31 patients with ESRD and 101 with normal renal function were matched from 47 ESRD patients in this study and 2439 in a previous cohort, respectively. All the patients had taken clopidogrel for more than 5 days before the platelet function were detected. The clinical characteristics of these are shown in Supplementary Table 1.

**Fig. 3:**
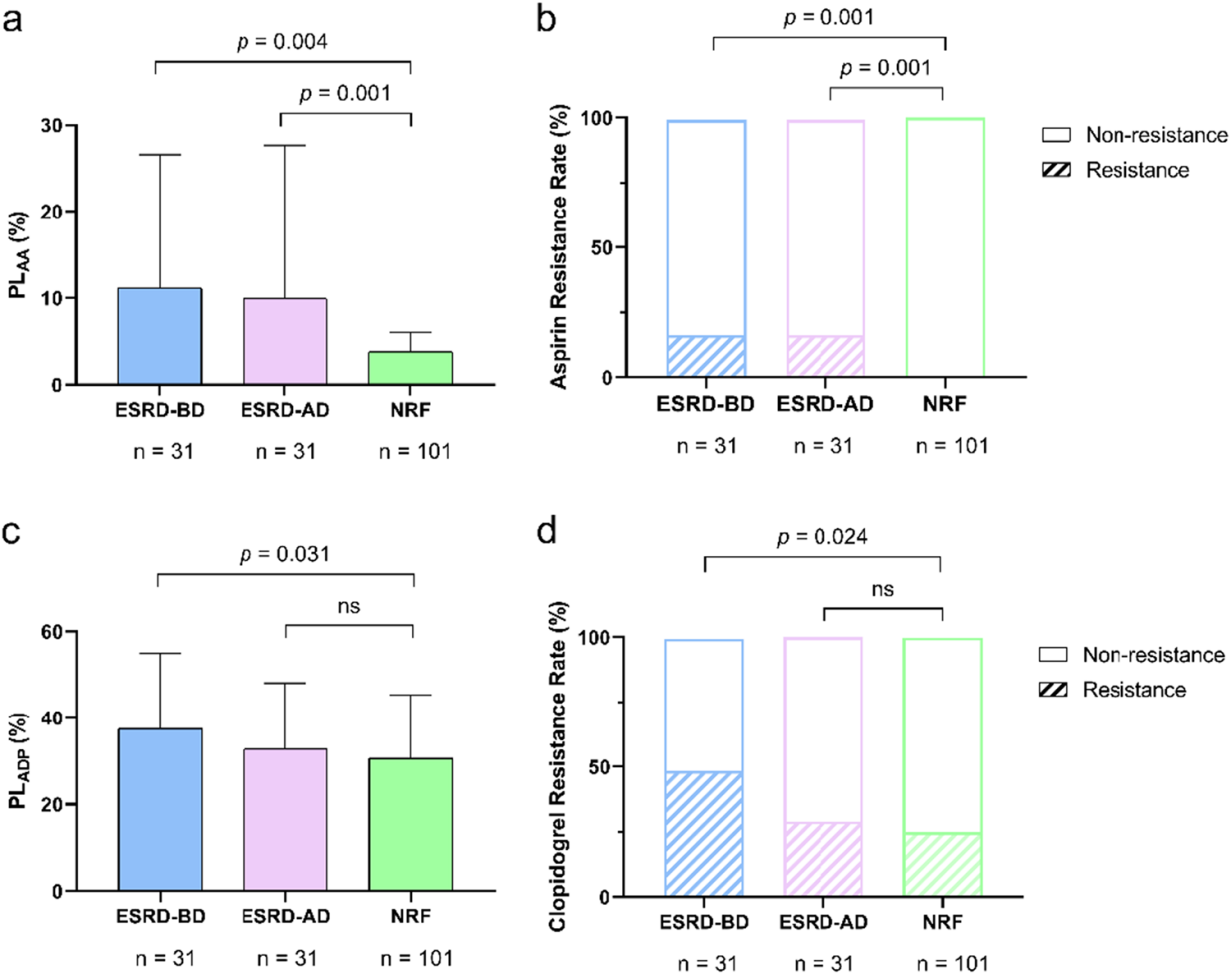
Platelet activities and the incidences of drug resistance in ESRD patients and those with NRF. **a** and **b** show the aspirin response and the incidence of aspirin resistance in ESRD patients and those with normal renal function. **c** and **d** show the clopidogrel response and the incidence of CR in ESRD patients and those with normal renal function; The striped portions represent the proportion of drug resistance. *ESRD* End-stage renal disease, *NRF* normal renal function, *ESRD-BD* patients with end-stage renal disease before dialysis, *ESRD-AD* patients with end-stage renal disease after dialysis, *CR* clopidogrel resistance, *PL*_*ADP*_ the maximum platelet aggregation rate induced by adenosine diphosphate, *PL*_*AA*_ the maximum platelet aggregation rate induced by arachidonic acid

PL_AA_ was significantly higher in ESRD patients both before and after hemodialysis compared to those with normal renal function (before dialysis: 11.1 ± 15.5 *vs.* 3.8 ± 2.2, *p* = 0.001; after dialysis: 9.9 ± 17.7 *vs.* 3.8 ± 2.2, *p* = 0.004) (Fig. 3a). Similarly, ESRD patients had a higher incidence of AR before and after hemodialysis compared to those with normal renal function (before hemodialysis: 16.1% *vs.* 0%, *p* = 0.001; after hemodialysis: 16.1% *vs.* 0%, *p* = 0.001) (Fig. 3b).

The PL_ADP_ was significantly higher in ESRD patients before hemodialysis compared to those with normal renal function (37.5 ± 17.4 *vs.* 30.6 ± 14.6, *p* = 0.031) (Fig. 3c), while PL_ADP_ after hemodialysis was not different from those with normal renal function (Fig. 3c).

Correspondingly, ESRD patients had a higher incidence of CR before hemodialysis compared to those with normal renal function (48.4% *vs.* 24.8%, *p* = 0.024) (Fig. 3d). While no significant difference was found in the incidence of CR after hemodialysis between ESRD patients and those with normal renal function (Fig. 3d).

## Discussion

In this study, we investigated the impact of hemodialysis on efficacies of the antiplatelet agents including aspirin, clopidogrel and ticagrelor in CAD patients complicated with ESRD. The main finding were as follows: (1) PL_AA_ remained unchanged after hemodialysis; (2) in patients taking clopidogrel, PL_ADP_ significantly decreased after hemodialysis, though PRU remained unchanged; Additionally, PL_ADP_ decreased after hemodialysis with polysulfone membrane, but not the polyamide membrane; (3) in patients taking ticagrelor, no significant change in PL_ADP_, or PRU was observed after hemodialysis; (4) AR and CR are more often presented in patients with CAD and ESRD compared to those with normal renal function.

The findings of this study indicate that hemodialysis does not diminish the efficacy of aspirin, clopidogrel, and ticagrelor, in patients with coronary artery disease (CAD) and might even enhance the efficacy of clopidogrel. The divergent outcomes observed between VerifyNow and LTA assays may be attributed to the limited sample size of patients subjected to VerifyNow testing.

Previous study results were controversial regarding the impact of hemodialysis on the efficacies of the antiplatelet agents. Patrik et al. detected the platelet function before and after hemodialysis in 31 CAD patients taking aspirin and clopidogrel using VerifyNow assay, in which they found that the antiplatelet effect of clopidogrel decreased after hemodialysis, while the effect of aspirin remained unchanged [12]. Geara et al. found that PRU decreased after hemodialysis in ESRD patients taking clopidogrel [13]. Fu et al. included 26 hemodialysis patients taking aspirin combined with clopidogrel after PCI and the patients’ platelet functions were monitored using thromboelastography. As a result, the antiplatelet effects of aspirin and clopidogrel remained unchanged after hemodialysis [20].

Aksu et al. reported that the incidence of AR was 51.9% before hemodialysis and 50.0% after hemodialysis in 54 patients using Multiplate tests [21]. By comparison, this study found that AR presented in 11.8% of the ESRD patients before hemodialysis, and 9.2% after hemodialysis by LTA assay. Though the incidences of AR differ between the two studies, both show no significant impact of hemodialysis on the antiplatelet effect of aspirin.

The effect of hemodialysis on platelet function is two-sided. It may activate platelets through artificial surfaces in hemodialysis pipelines and extrusion of peristaltic pumps during hemodialysis [22]. It may also affect the cytoskeleton of platelets and affect downstream signal transduction pathways, or remove toxins in patients to improve platelet response to the antiplatelet agents [23, 24]. This may explain the inconsistent results between different studies. The different hemodialysis membranes may have impact on the efficacies of the antiplatelet agents but rarely investigated. Patrik et al. found an increase in CR following hemodialysis with polyamide membrane, whereas polysulfone membrane did not change the incidence of CR [12]. On the contrary, we found that the polysulfone membrane led to lower PL_ADP_ levels after hemodialysis compared to the polyamide membrane in the ESRD patients taking clopidogrel. This effect was not observed with the polyamide membrane, suggesting that the polysulfone membrane may enhance the efficacy of clopidogrel.

Consistent with previous studies [12, 25], higher incidence of CR in ESRD patients compared to those with normal renal function has been found in our study. The incidence of CR in patients with ESRD was about doubled that with normal renal function (Table 2). However, the incidence of CR after hemodialysis was not statistically different from those with normal renal function. This result further indicated that hemodialysis significantly improved the antiplatelet effect of clopidogrel.

It has been reported that the antiplatelet effect of ticagrelor is superior to clopidogrel in ESRD patients [26]. Consistent with previous study, we found that the platelet aggregation was ideally inhibited in ESRD patients treated with ticagrelor, showing very rare incidence of ticagrelor resistance. However, those allocated to clopidogrel presented high incidence of CR. Summaria et al. replaced clopidogrel to ticagrelor in hemodialysis patients with high platelet reactivity, and found that about 90% of the patients changed from high platelet reactivity to low platelet reactivity [27]. That result along with ours favors the use of ticagrelor in ESRD patients if the thrombosis risk is prevailing.

Fujii et al. reported that, in patients with acute coronary syndrome, the probability of bleeding was positive correlated with renal dysfunction, regardless of the dual antiplatelet treatment (DAPT) regimen used [28]. This probability was consistently higher in clopidogrel than in prasugrel, and this trend was also shown in maintenance hemodialysis patients, suggesting that prasugrel is safer than clopidogrel as a component of DAPT throughout all levels of renal function, including hemodialysis patients after ACS [28]. Thus, in areas where ticagrelor is not available, prasugrel would be an alternative option for patients taking DAPT and requiring hemodialysis due to ESRD.

## Strengths and limitations

Our study used different platelet function assays to comprehensively evaluate the impact of hemodialysis on the efficacies of aspirin, clopidogrel, and ticagrelor under different hemodialysis membranes. There are two limitations of this study: (1) Due to the heterogeneity of the internal environment between patients with ESRD and those with normal renal function, it was not feasible to achieve a successful match of four patients with normal renal function for every ESRD patient in the propensity matching process. However, from this exploratory research, we proved that hemodialysis does not affect the antiplatelet effect of aspirin and ticagrelor, though the impact of hemodialysis on clopidogrel response need to be further approved. (2) We did not record the clinical events in this study. As the primary purpose of this study was to investigate the impact of hemodialysis on efficacies of the antiplatelet agents, and we would not expect a meaningful difference regarding either thrombotic or bleeding events between the clopidogrel and ticagrelor groups in this small sample size study.

## Conclusion

Hemodialysis does not have negative effect on the efficacies of aspirin, clopidogrel and ticagrelor in ESRD patients with CAD. ESRD patients have higher incidences of AR and CR compared with those with normal renal function.

### Supplementary Information

Below is the link to the electronic supplementary material.

Supplementary file1 (DOCX 17 KB)

## Data Availability

All data and materials are available from the corresponding authors upon written request.
