# Supplementary material for: Impact of hemodialysis on efficacies of the antiplatelet agents in coronary artery disease patients complicated with end-stage renal disease
Source: J Thromb Thrombolysis. 2024 Feb 23;57(4):558–65. doi: 10.1007/s11239-023-02924-5 (PMC11026285; doi:10.1007/s11239-023-02924-5)
Supplement: Supplementary file 1 — Supplementary file1 (DOCX 17 KB) [file 11239_2023_2924_MOESM1_ESM.docx]

**Supplementary table 1** Baseline characteristics of CAD patients with ESRD or normal renal function

| Characteristics | ESRD  n = 31 | NRF  n = 101 | *p*-value |
| --- | --- | --- | --- |
| Age (years) | 69.8 ± 10.9 | 69.6 ± 9.3 | 0.927 |
| Gender (%) | 0(0%) | 5(3.8%) | 0.591 |
| BMI (kg/m2) | 24.6 ± 3.2 | 24.5 ± 2.8 | 0.830 |
| Smoking (%) | 15(48.4%) | 47(46.5%) | 1.000 |
| Hypertension (%) | 27(87.1%) | 89(88.1%) | 1.000 |
| Diabetes (%) | 21(67.7%) | 59(58.4%) | 0.405 |
| Statins (%) | 28(90.3%) | 96(95%) | 0.390 |
| PPIs (%) | 16(51.6%) | 51(50.5%) | 1.000 |
| Uric acid (μmol/L) | 311.8 ± 128.2 | 347.1 ± 101.3 | 0.119 |

Values are presented as n (%) or mean ± SD

*CAD* Coronary heart disease; *ESRD* End-stage renal disease; *NRF* Normal renal function; *BMI* Body mass index; *PPIs* Proton pump inhibitors
